# Precision medicine in schizophrenia: stratifying patients by inflammatory, metabolic, and gut-brain biomarker profiles using latent profile analysis

**DOI:** 10.1192/j.eurpsy.2026.10536

**Published:** 2026-07-31

**Authors:** M. Couce-Sanchez, F. Dal Santo, M. Bernardo, G. Safont, M. P. Garcia-Portilla, B. Arranz, L. Gonzalez-Blanco

**Affiliations:** 1 SESPA - AGC Salud Mental AS IV. Área de Psiquiatría - Universidad de Oviedo. Grupo de Investigación en Psiquiatría - ISPA. INEUROPA. G05 - CIBERSAM, Oviedo; 2 Barcelona Clinic Schizophrenia Unit, Hospital Clinic, Departament de Medicina, Institut de Neurociències (UBNeuro), Universitat de Barcelona (UB). Institut d’Investigacions Biomèdiques August Pi I Sunyer (IDIBAPS), ISCIII. CIBERSAM; 3 Department of Psychiatry, Hospital Universitari Mútua Terrassa, ISIC Medical Center. University of Barcelona. CIBERSAM; 4 Parc Sanitari Sant Joan de Deu. CIBERSAM, Barcelona, Spain

## Abstract

**Introduction:**

Schizophrenia is a heterogeneous mental disorder with diverse clinical and biological profiles. Current diagnostic systems relying solely on symptoms may overlook the full diversity of patients’ presentations, so developing neurobiological subtypes has been of great interest in recent research (Clementz et al. Schizophr Bull 2022; 48 56-68).

Biomarkers such as inflammatory markers (Lalousis et al. Brain Behav Immun 2023; 113 166-175), features of metabolic syndrome (Saccaro et al. Front Psychiatry 2024; 15 1225693), and gut-brain axis indicators (González-Blanco et al. Eur Psychiatry 2024; 67(1) e84) provide insights into pathophysiology and could enhance precision psychiatry approaches.

**Objectives:**

To stratify schizophrenia patients into subgroups based on inflammatory, metabolic, and gut-brain biomarkers. To investigate associations of such profiles with clinical outcomes, including symptom severity and cognitive performance.

**Methods:**

Data from 199 patients diagnosed with schizophrenia (DSM-5) (mean age 40.9 years, 60.3% male), enrolled in an observational, cross-sectional, multicentre study from four centres in Spain (PI17/00246) were analysed.

Assessment:

Biomarkers:

Inflammatory: C-reactive protein (CRP; mg/L).

Metabolic: Abdominal circumference (cm)

Gut-brain axis: Lipopolysaccharide-binding protein (LBP; μg/mL).

Psychometrics: CGI-SCH, Screen for Cognitive Impairment in Psychiatry (SCIP), Positive and Negative Syndrome Scale (PANSS) for Schizophrenia.

Statistics:

Models based on latent profile analysis (LPA) with 1 to 6 profiles were estimated and compared using AIC, BIC, entropy, and the bootstrap likelihood ratio test (BLRT).

Differences in cognitive tests (SCIP) and symptom severity (PANSS, CGI-SCH) were assessed by ANCOVA (controlled by age). Analyses were conducted in R.4.5.1 (package: tidyLPA)

**Results:**

A two-profile solution (AIC = 1555, BIC = 1588, entropy = 0.930, BLRT < 0.01) was selected as optimal for balancing fit, parsimony, and clinical interpretability. Two distinct biomarker profiles emerged: a normative profile (91.5% of participants) with typical biomarker levels, and a high-risk profile (8.5%) with elevated inflammatory and metabolic markers (see profiles’ representation in **image 1**).

The high-risk group showed significant impairments in working memory and verbal fluency compared to the normative group (see comparison in **figure 2**); no significant differences were found in PANSS or CGI-SCH scores.

**Image 1:**
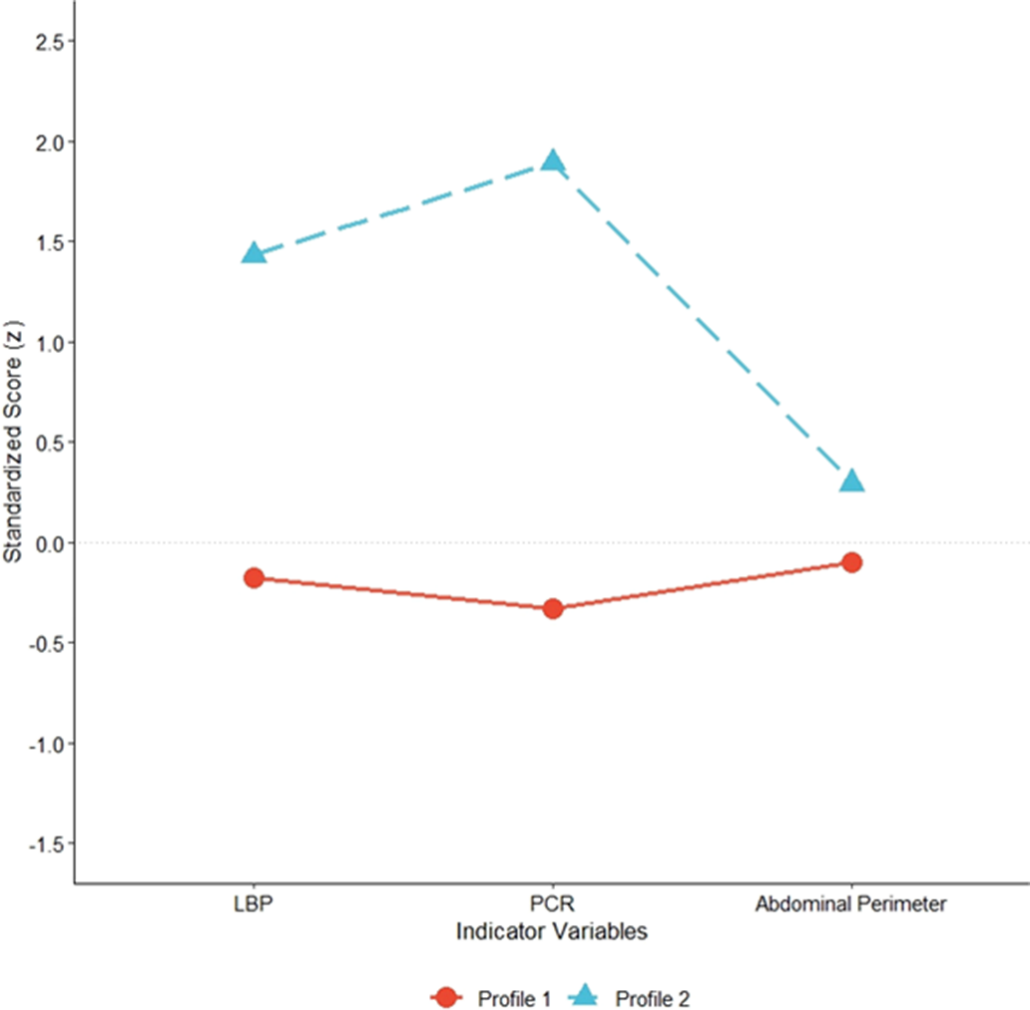
Image 1: Long description.

**Image 2:**
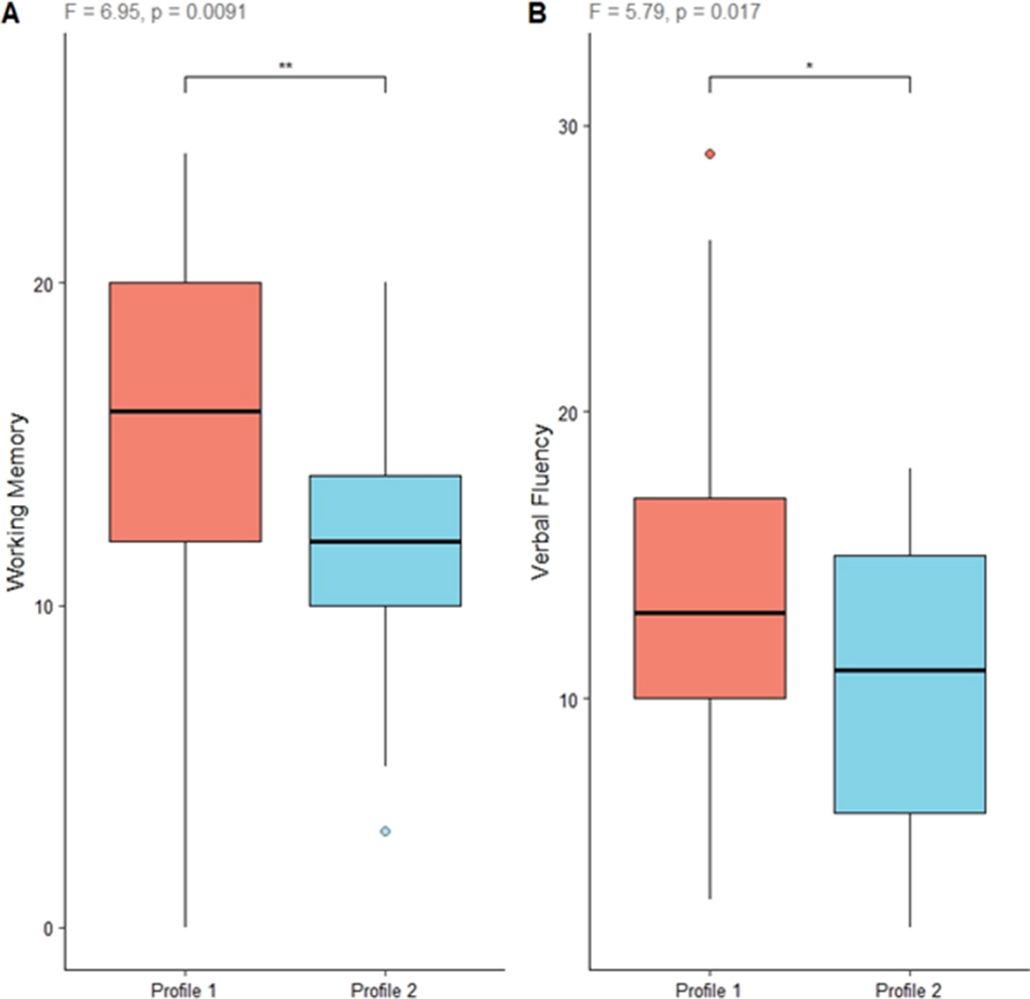
Image 2: Long description.

**Conclusions:**

Integrating inflammatory, metabolic, and gut-brain biomarkers via LPA identifies distinct schizophrenia patient subgroups. The high-risk profile is associated with cognitive deficits despite comparable symptom severity. These findings support biomarker-informed precision psychiatry for targeted treatment approaches.

**Disclosure of Interest:**

None Declared

## Long descriptions

### Image 1: Long description

The Y-axis is labeled Standardized Score (z) and ranges from -1.5 to 2.5 in increments of 0.5, with a horizontal dotted line at 0.0. The X-axis is labeled Indicator Variables and includes three categories: L B P, P C R, and Abdominal Perimeter.

* Profile 1 (red solid line with circles): This profile remains below the 0.0 baseline for all variables. It starts at approximately -0.2 for L B P, dips slightly to -0.3 for P C R, and rises back to -0.1 for Abdominal Perimeter.

* Profile 2 (blue dashed line with triangles): This profile remains above the 0.0 baseline. It starts at approximately 1.4 for L B P, peaks at 1.9 for P C R, and drops sharply to 0.3 for Abdominal Perimeter.

A legend at the bottom identifies Profile 1 with a red circle and Profile 2 with a blue triangle.

Navigate back to Image 1:.

### Image 2: Long description

Two vertical box plots labeled A and B.

Panel A: Working Memory

- Statistical header: F = 6.95, p = 0.0091.

- Y-axis: Working Memory, ranging from 0 to 30.

- X-axis: Profile 1 (salmon pink) and Profile 2 (light blue).

- Profile 1: Median is approximately 15. The box spans from 12 to 20. Whiskers extend from 0 to 26.

- Profile 2: Median is approximately 12. The box spans from 10 to 14. Whiskers extend from 6 to 20. A single outlier dot is located near 4.

- A bracket with two asterisks indicates a significant difference between the two profiles.

Panel B: Verbal Fluency

- Statistical header: F = 5.79, p = 0.017.

- Y-axis: Verbal Fluency, ranging from 0 to 30.

- X-axis: Profile 1 (salmon pink) and Profile 2 (light blue).

- Profile 1: Median is approximately 13. The box spans from 10 to 17. Whiskers extend from 4 to 24. A single red outlier dot is located near 29.

- Profile 2: Median is approximately 11. The box spans from 6 to 14. Whiskers extend from 0 to 18.

- A bracket with one asterisk indicates a significant difference between the two profiles.

Navigate back to Image 2:.

